# Tasteless Preparations of Cascara Sagrada

**Published:** 1888-03

**Authors:** 


### *TASTELESS PREPARATIONS OF CASCARA SAGRADA (RHAMNUS PURSHIANA).*

(Abstract of an article entitled "An Examination of Cascara Sagrada," by H. F. MEIER and J. LEROY WEBBER, published in the *American Journal of Pharmacy*, February, 1888.)

Recent investigation of the constituents of Cascara sagrada has led to the discovery of new principles and facts of great importance pharmaceutically and therapeutically.

The chief objection to Cascara sagrada heretofore has been its inherent bitterness. In the light of recent researches, tasteless preparations of this drug, highly efficacious medicinally, are now to be had.

These discoveries mark a distinct advance in pharmacal attainment and in the therapeutics of chronic constipation, since this remedy can now be much more generally and persistently administered, and its well-known tonic laxative action obtained without the drawbacks which seemed formerly inseparable from its employment.

The facts disclosed concerning this remedy deserve more than a passing notice, especially since they indicate the existence of principles and modes of action extending far beyond the subject indicated, and are well worth the close attention of the thoughtful and scientific physician. A valuable contribution to the knowledge of the chemical constitution of this drug appeared in the *Amer. Journal of Pharmacy*, for February, 1888, which makes it possible not only to obtain a true interpretation of the various clinical observations, but clears up apparent anomalies, and also indicates the reasons for observed effects, which have lately been disputed, but now admit of no further question or misunderstanding.

Among the discoveries referred to in this valuable paper, of especial interest to the physician, is the influence of a class of vegetable ferments, and their recognition as the causes of various abnormal conditions, such as colic, vomiting, nausea, diarrhœa and dysentery, which occasionally attend the administration of certain drugs.

It appears that Frangula bark, when fresh, contains such a ferment in excessive quantities, and is, therefore, unfit for use until the ferment has exhausted itself—the process usually occupying several years. It also appears that Cascara contains some of this principle, and this fact will account for the occasional untoward effects of the drug, which have been observed as consequent on the employment of a number of its preparations heretofore in the market. These effects are, therefore, not due, as has been supposed, to any idiosyncrasy on the part of the patient, or to the laxative or tonic constituents of the bark itself, but to a distinct objectionable principle, which, once recognized, can be rendered inoperative and harmless.

It has been reserved for Parke, Davis & Co., through their exhaustive investigations, to be the first to clearly recognize the principles involved, and by the application of such intelligent comprehension, to formulate and adopt correct pharmaceutical processes, and thus overcome all the difficulties heretofore existing. As a result of their investigations, they now offer to the medical profession a fluid extract, a solid extract and also a concentration, all of which (designated as "Formula of 1887") exhibit only the desirable laxative and tonic properties, and being free from this ferment, are incapable of producing griping, nausea or any of the mal-effects above enumerated.

It appears that these ferments are distributed through a large number of vegetable substances, being not confined to unripe fruits only, but can also exist in the root, bark, leaf or even in vegetable extracts, of which we have illustrations in various juices, liquid or inspissated. Of this latter class, aloes will serve for an example. A familiar illustration of an unaltered vegetable would be the cucumber, the green apple (familiar to the school-boy), and unripe fruit generally. In the case of the cucumber, experience has taught the means of removing this ferment by dialysis or osmosis. We sprinkle salt over it or surround it with a strong brine, which provokes an outward flow of the fluid containing the ferment, with the result that the ferment is to a large extent removed, and thus rendered incapable of producing the same conditions in the stomach for which it was intended in the plant; that is, the creation of vegetable acids from other material previously existing, in the same manner that pepsin, likewise an unorganized and soluble ferment, provokes the solution of fibrin and albumen, forming peptone, or, as diastase, is capable of effecting the transformation of starch into soluble glucose and dextrin, both new bodies.

That these ferments all bear a direct quantitative proportion to the results accomplished, has been practically recognized. We are promised a satisfactory indication of the sources of the acids formed in the plant, which will enable us to corroborate the statements that identical processes go on in the stomach when the ferment is permitted to exert its action there.

The physiological tests now being conducted at the laboratory of Parke, Davis & Co., with the different principles contained in the plant, cannot fail to demonstrate finally not only the superiority of Cascara itself, to its former supposed competitor, Frangula, but also its comparative value as a laxative.

To physicians desiring fuller information concerning the discoveries made, a reprint of the article from the *American Journal of Pharmacy*, and a working bulletin descriptive of this drug, will be mailed by Parke, Davis & Co., free on request.

